# A Systematic Review and Meta-Analysis of Dengue Risk with Temperature Change

**DOI:** 10.3390/ijerph120100001

**Published:** 2014-12-23

**Authors:** Jingchun Fan, Wanxia Wei, Zhenggang Bai, Chunling Fan, Shulan Li, Qiyong Liu, Kehu Yang

**Affiliations:** 1 First Clinical Medical College, Lanzhou University, No. 1 Donggang West Road, Chengguan District, Lanzhou, Gansu 730000, China; E-Mail: fan_jc@126.com; 2 Key Laboratory of Evidence Based Medicine and Knowledge Translation of Gansu Province, No. 222 Tianshui South Road, Chengguan District, Lanzhou, Gansu 730000, China; E-Mail: baizhenggang@126.com; 3 Evidence-Based Medicine Center, School of Basic Medical Sciences, Lanzhou University, No. 222 Tianshui South Road, Chengguan District, Lanzhou, Gansu 730000, China; 4 State Key Laboratory for Infectious Disease Prevention and Control, National Institute for Communicable Disease Control and Prevention, China CDC, No. 155 Changbai Road, Changping District, Beijing 102206, China; 5 University Hospital of Gansu Traditional Chinese Medicine, No. 732 Jiayuguan West Road, Chenguang District, Lanzhou, Gansu 730020, China; E-Mail: wanxia_wei@sina.com; 6 Department of Clinical Pharmacy, Gansu Provincial Cancer Hospital, No. 2 Xiaoxihu East Street, Qilihe District, Lanzhou, Gansu 730050, China; E-Mail: fanchl8@sina.com; 7 Department of Ultrasound, People’s Hospital of Gansu Province, No. 204 Donggang West Road, Chengguan District, Lanzhou, Gansu 730000, China; E-Mail: vivian138lan@163.com; 8 Collaborative Innovation Center for Diagnosis and Treatment of Infectious Diseases, No. 866 Yuhangtang Road, Xihu District, Hangzhou, Zhejiang 310058, China

**Keywords:** dengue fever (DF), temperature, correlation, odds ratio (OR), systematic review, meta-analysis

## Abstract

Dengue fever (DF) is the most serious mosquito-borne viral disease in the world and is significantly affected by temperature. Although associations between DF and temperatures have been reported repeatedly, conclusions have been inconsistent. Six databases were searched up to 23 March 2014, without language and geographical restrictions. The articles that studied the correlations between temperatures and dengue were selected, and a random-effects model was used to calculate the pooled odds ratio and 95% confidence intervals. Of 1589 identified articles, 137 were reviewed further, with 33 satisfying inclusion criteria. The closest associations were observed between mean temperature from the included studies (23.2–27.7 °C) and DF (OR 35.0% per 1 °C; 95% CI 18.3%–51.6%) positively. Additionally, minimum (18.1–24.2 °C) (29.5% per 1 °C; 20.9%–38.1%) and maximum temperature (28.0–34.5 °C) (28.9%; 10.3%–47.5%) were also associated with increased dengue transmission. The OR of DF incidence increased steeply from 22 °C to 29 °C, suggesting an inflexion of DF risk between these lower and upper limits of DF risk. This discovery is helpful for government decision-makers focused on preventing and controlling dengue in areas with temperatures within this range.

## 1. Introduction

Dengue fever (DF) has been ranked as the most serious mosquito-borne viral disease in the world since 2012 [1], and has a higher morbidity and more severe economic impact than malaria [2]. The global population living in a dengue risk area has been estimated to have increased from 30% to 54.7% (2.05–3.74 billion)_ENREF_3, and the WHO has reported that 230 million new infections occur each year, including over 2 million with severe symptoms, and 21,000 deaths from DF in more than 119 tropical or subtropical countries across five continents [3]. Dengue infection is a systemic and dynamic disease transmitted between humans by *Aedes* mosquitoes [3,4], and it ranges widely in the clinical spectrum from asymptomatic or self-limiting to severe clinical manifestations. Sometimes dengue hemorrhagic fever (DHF) and dengue shock syndrome (DSS) occur [5]. The epidemiological triangle of DF includes susceptible populations, dengue viruses, mosquito vectors (including *Aedes aegypti* and *Aedes albopictus*) together with their interactions in the environment [6]. The dengue virus would complete part of its development in the mosquito vectors which were sensitive to environmental factors, so that dengue transmission is sensitive to climate. [7]. The principal vectors of *Aedes* mosquitoes are poikilothermic creatures whose developmental period of life cycle and parasites developed in their bodies are directly influenced by climatic conditions [8], especially temperature. As the temperature rises, DF transmission accelerates due to the increasing development rate of mosquito and shortening virus incubation time in the body of the mosquitoes [9]. The Watts experiments in Bangkok demonstrated that a higher incubation temperature varying from 20 °C to 35 °C would accelerate the rate of virus transmission by *Aedes aegypti* [10]. Oviposition declined steeply, however, if the monthly mean temperature fell to 16.5 °C, and no eggs were produced if the mean temperature decreased to 14.8 °C in Buenos Aires [11]. Minimum temperature was critical in many regions for mosquito survival, development rates, and sustaining the population density [12,13]. Low temperatures adversely were found to affect the survival of adult and immature *Aedes* mosquitoes, whereas historically high minimum temperatures might assist larval survival in winter [14]. It has been reported that the critical minimum temperature suitable for DF transmission throughout the year was 21 °C when the daily survival rate of *Aedes Agypti* was 0.89 [15]. However, many studies have claimed that temperature is not correlated with dengue transmission, such as in the DF outbreak in Cixi, Zhejiang Province, China, in 2009. That epidemiological investigation found that the incidence of DF was not associated with temperature or other climate factors [16]. Precipitation had a statistically significant connection with the incidence of DF, whereas temperatures were not associated with DF, in Medellin, Colombia [17].

Moreover, there is extensive observational evidence showing that warming of the climate system is unequivocal. The surface temperatures at high altitude areas have demonstrated more sensitive and vulnerable warming trends than have those at low elevations [18,19,20]. According to the International Council of Scientific Unions and the Intergovernmental Panel on Climate Change (IPCC) a potential expansion is expected to occur in the latitudinal and altitudinal range of dengue by the end of the next century based on the current warming projection [21]. In temperate zone, the potential range of the transmission season will also expand. [22]. A slight rise in temperatures is potentially-associated with a substantial increase in dengue outbreaks [6]. Given this risk, we comprehensively reviewed the evidence concerning the relationship between temperatures and dengue risk using a systematic review and meta-analysis to explore the trend and extent of dengue risk -due to temperature changes and provide evidence for dengue prevention and control.

## 2. Methods

### 2.1. Search Strategy and Selection Criteria

We systematically searched PubMed, Embase, Web of Science, Biosis Previews, Cochrane library, and China biomedical literature database to obtain the studies on the associations of temperature with dengue transmission. We used the following keywords: “dengue”, “dengue fever”, “dengue hemorrhagic fever”, “dengue shock syndrome”, “DF”, “DHS”, “DSS”, “temperature”, “temperatures” and “temp”. We considered studies that reported connections between temperature and dengue incidence or dengue cases, there were no geographical or language restrictions, and we included only peer-reviewed original articles. Bibliographic reference lists of studies selected by inclusion criteria in our meta-analysis and relevant review articles were manually searched (Appendix). We limited the databases of our search from inception to 23 March 2014.

### 2.2. Data Extraction

Data were extracted and reviewed independently by Jingchun Fan, Weixia Wan and Zhenggang Bai, with disagreements adjudicated via discussion among three authors (Weixia Wan, Zhengang Bai and Chunling Fan), and compiled with a standardized data collection form in duplicate. We contacted authors of the included studies for additional data or clarification as needed. Jingchun Fan and Weixia Wan retrieved titles and abstracts initially and then screened the relevant full-text articles. Zhenggang Bai and Chunling Fan recorded the following characteristics in the included studies: first author, publication year, studied location, the altitude, climatic zone, studied period, data source of dengue, data source of meteorology, statistical method, studied variable (*T_min_,* *T_max__,_ T_mean_*), dengue incidence or case, and the unadjusted effect sizes with 95% CI.

### 2.3. Quality Assessment

Quality assessment was independently performed by two authors (Jingchun Fan and Wanxia Wei). The quality of selected studies was assessed using the combined criteria suggested by Pai *et al.* [23], Wells *et al.* [24] and Shah *et al.* [25]. As a result, the quality of each study included in the meta analysis was determined across ten metrics: generalizability, description of temperature, dengue cases or incidence, source of dengue data, reporting bias, limitation, multiple lag, adjusted for time trend, adjusted for seasonality and fund supporting.

### 2.4. Statistical Analysis

Studies that met the eligibility criteria and that reported unadjusted or adjusted odds ratios (OR) with 95% CI, or presented sufficient data for the calculation of unadjusted OR and 95% CI, were included in the meta-analysis. We calculated the standardized risk estimates one by one for each study using the following formula for a standardized increment in 1 °C, it means if the temperature increases/decreases 1 °C, the degree of OR and 95% confidence intervals will increase/decrease:

$$OR_{\left(standardised\right)}=OR_{\left(original\right)}^{increment\;1\;℃/\text{increment}\left(\text{original}\right)}$$

The pooled ORs and 95% CIs were calculated using I-V heterogeneity method and random-effects model. We anticipated heterogeneity between studies due to different methods of analysis, different lag exposures, climatic zone, and altitude by meta regression and subgroup, and the number of the Monte Carlo permutation test was set to 10,000. A random-effects model was used to account for both within and between study heterogeneity. We produced forest plots to visually assess the OR and 95% CI of each study, and used funnel plots to assess publication bias (with study size as a function of effect size). For studies that only reported *p*-values, the Fisher’s *χ^2^* test was used to compare the combined *p*-values. We used Egger’s linear regression method to test for funnel plot asymmetry (*i.e.*, to quantify the bias captured by the funnel plot). We considered the presence of heterogeneity at a 10% level of significance and *I^2^* exceeding 50%. Analysis was performed using Stata Software (Version 12.0, StataCorp, TX, USA). Statistical significance was taken as two-sided *p <* 0.05.

## 3. Results

### 3.1. Study Inclusions

The initial search yielded 1589 records, of which 137 remained after removal of duplicates and screening of titles and abstracts, and 33 records strictly met the inclusion criteria [12,14,16,17,26,27,28,29,30,31,32,33,34,35,36,37,38,39,40,41,42,43,44,45,46,47,48,49,50,51,52,53,54] (the full search criteria are shown in the Supplementary File Figure S1). Twenty-three articles included minimum temperature analysis [14,16,17,26,27,28,29,31,33,34,35,36,37,38,40,42,43,47,49,50,52,53,54], fourteen articles included maximum temperature analysis [16,17,26,27,28,31,33,36,38,40,43,45,46,47], and sixteen articles studied the association between mean temperature and dengue transmission [12,16,17,26,27,28,30,32,33,37,39,41,43,44,48,51]. One article was excluded from the meta-analysis because the OR could not be calculated from the available data (n = 3) [55]. Multiple linear regression and multivariate Poisson regression were the two main statistical analyses used to associate dengue and temperatures. Most locations of the included studies were in tropical regions of eastern Asia, southeast Asia and Latin America (Supplementary File Figure S2), and the elevations were less than 1000 m, except for Medellin, Colombia [17]. Baseline characteristics of the included studies are available in the Supplementary File Table S1.

In terms of the quality of included studies, agreement between the two reviewers was 92% (Cohen’s kappa = 0.852). Five studies scored full points (10); the range of total points of included studies was four to ten (Supplementary File Table S2).

### 3.2. The Association Analysis between Temperatures and OR of Dengue

We extracted the data of temperatures, OR and 95% CI to illustrate the relationship between temperatures and dengue transmission. The minimum temperatures of included articles varied from 18.1 °C to 24.2 °C, the maximum temperatures ranged from 28.0 °C to 34.5 °C, and the mean temperatures ranged from 23.2 °C to 27.7 °C.

**Figure 1:**
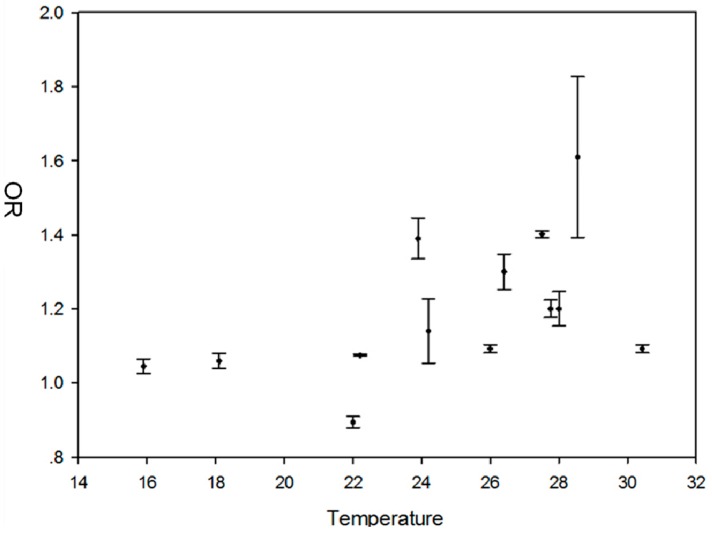
The scatter plot between extracted temperatures and OR of DF.

Figure 1 shows that from approximately 16 °C to 22 °C, the impact of temperature on dengue risk remains at a lower level; from approximately 22 °C, the OR of dengue risk increases steeply up to 29 °C; and that dengue risk begins to decrease if temperatures are higher than 29 °C.

According to the meta-analysis of temperatures and dengue incidence or cases, there was a positive association between DF cases or incidence and temperature, including minimum temperature, maximum temperature and mean temperature (Table 1). The strongest associations were observed between mean temperature and dengue (OR 35.0% per 1 °C; 95% CI 18.3%–51.6%). The minimum temperature (OR 29.5% per 1 °C; 95% CI 20.9%–38.1%) and maximum temperature (28.9% per 1 °C; 95% CI 10.3%–47.5%) were also positively correlated with dengue incidence or cases.

Although a random-effects model was used to analyze the heterogeneity between studies and substantial overlap between CI for the results, high *I^2^* values and *p* < 0.05 suggested substantial heterogeneity between studies (the mean temperatures, minimum temperatures and maximum temperatures). We performed a meta regression with Monte Carlo permutation test to explore the factors of heterogeneity, and the results demonstrated that lag exposures, altitudes, climatic zone and the various methods adopted in the included articles were not the factors that contributed to heterogeneity in our study (Table 2).

**Table 1:** OR and 95% CI between *T_min_*, *T_max_* and *T_mean_* and DF risk in meta-analysis (Random-effects model).

Study	OR	95% CI	
*T_min_*			
Promprou *et al.* [27]	1.08	1.08	1.08
Hurtado-Daiz *et al.* [29]	1.04	1.00	1.08
Hurtado-Daiz *et al.* [29]	1.06	1.02	1.10
Chowell *et al.* [28]	1.62	1.53	1.71
Hsieh *et al.* [33]	1.19	0.96	1.48
Lu *et al.* [14]	1.42	1.26	1.57
Lu *et al.* [14]	1.37	1.23	1.51
Chen *et al.* [34]	1.15	1.12	1.18
Chen *et al.* [34]	1.71	1.67	1.75
Sriprom *et al.* [35]	2.69	2.44	2.97
Colon- González *et al.* [36]	1.08	1.03	1.13
Gharbi *et al.* [37]	1.11	0.98	1.27
Pinto *et al.* [40]	1.3	1.2	1.45
Gomes *et al.* [42]	1.45	1.34	1.58
Gomes *et al.* [42]	0.89	0.87	0.92
Cheong *et al.* [47]	1.14	1.04	1.46
Huang *et al.* [49]	1.64	1.01	2.67
Li *et al.* [50]	1.10	1.08	1.12
Ibarra *et al.* [52]	1.02	0.98	1.09
Fan *et al.* [53]	1.83	1.50	2.17
Wang *et al.* [54]	0.84	0.80	0.88
Overall	1.30	1.21	1.38
*T_mean_*			
Promprou *et al.* [27]	1.40	1.39	1.44
Chowell *et al.* [28]	2.84	2.66	2.96
Arcari *et al.* [30]	1.49	1.38	1.60
Hsieh *et al.* [33]	1.36	1.21	1.52
Wu *et al.* [12]	1.95	1.32	2.58
Gharbi *et al.* [37]	1.26	1.03	1.53
Pham *et al.* [39]	1.39	1.25	1.55
Earnest *et al.* [41]	1.20	1.14	1.25
Hii *et al.* [44]	1.31	1.22	1.40
Hii *et al.* [44]	1.07	1.00	1.15
Hii *et al.* [44]	1.46	1.36	1.56
Hii *et al.* [44]	1.39	1.30	1.47
Goto *et al.* [48]	1.18	1.04	1.8
Goto *et al.* [48]	1.00	0.99	1.01
Goto *et al.* [48]	1.64	1.36	1.92
Lowe *et al.* [51]	1.65	1.55	1.79
Overall	1.35	1.18	1.52
*T_max_*			
Chowell *et al.* [28]	2.00	1.89	2.11
Brunkard *et al.* [31]	1.03	1.00	1.05
Hsieh *et al.* [33]	2.33	1.89	2.42
Colon- González *et al.* [36]	1.13	0.99	1.27
Pinto *et al.* [40]	1.20	1.11	1.33
Gomes *et al.* [42]	1.09	1.07	1.12
Hu *et al.* [45]	1.61	1.03	2.41
Karim *et al.* [46]	1.01	0.93	1.10
Karim *et al.* [46]	1.09	1.01	1.17
Karim *et al.* [46]	1.14	1.07	1.21
Overall	1.29	1.10	1.48

**Table 2:** Meta regression analysis on the factors and OR of dengue.

Temperatures *p*		Lag	Altitude	Method	Climatic Zone	Largest Monte Carlo SE (*p*)
*Tmin*	Unadjusted *p*	0.889	0.500	0.667	0.889	0.1034
	Adjusted *p*	1.000	1.000	1.000	1.000	
*Tmax*	Unadjusted *p*	0.333	0.667	1.000	0.333	0.2191
	Adjusted *p*	1.000	1.000	1.000	1.000	
*Tmean*	Unadjusted *p*	0.822	0.592	0.557	--	0.0050
	Adjusted *p*	0.991	0.933	0.894	--	

Note: “--” Climatic zone removed because of collinearity.

### 3.3. Meta-Analysis of p-Values and Publication Bias Test

When we combined *P* values from the articles that did not have sufficient data to calculate OR and 95% CI, the results showed a positive relationship between minimum temperature, maximum temperature and mean temperature and the dengue incidence or cases (Table 3). According to Egger’s publication bias test, we did not find publication bias in the included articles (*p* > 0.05, Table 4, and Supplementary File Figure S3).

**Table 3:** Combined *p*-values for temperatures and dengue.

Temperatures	Studies	χ^2^	*p*_Value	Two-Tailed *p*
*Tmin*	7 [16,17,26,27,31,38,43]	33.591478	0.00021655	0.000433
*Tmax*	7 [16,17,26,27,38,43,47]	53.472649	6.321 e^−6^	1.26 e^−5^
*Tmean*	6 [16,17,26,27,32,43,]	29.511036	0.00025784	0.000516

**Table 4:** Egger’s test for publication bias.

Temperatures	Statistic	Coef.	Std. Err.	*t*	*p*	95% CI	
*Tmin*	Slope	0.0695253	0.030069	2.31	0.034	0.00608	0.13296
	bias	4.9533650	2.930330	1.69	0.109	−1.22903	11.13576
*Tmax*	Slope	0.0407122	0.123009	0.33	0.750	−0.25015	0.33158
	bias	3.8721170	4.255641	0.91	0.392	−6.19087	13.93511
*Tmean*	Slope	0.3523181	0.088274	3.99	0.001	0.16288	0.54164
	bias	−0.7215385	3.319929	−0.22	0.831	−7.84207	6.39934

## 4. Discussion

In this study, we first presented a meta-analysis of the worldwide correlation between dengue incidence and temperatures and provided the temperature conditions most conducive for dengue transmission. Robust and clear connections between temperature (mean temperature, maximum temperature and minimum temperature) and dengue incidence or cases were evident, especially mean temperature (23.2 °C–27.7 °C). All studies included in this review were conducted in tropical or subtropical areas in developing countries, except Australia and Singapore, which agrees with dengue virus distribution in the world [56].

It is well known that temperature positively affects dengue incidence, and the impact of temperature on dengue transmission has been repeatedly reported in various regions, but the relationships between temperature and dengue is significant in some studies but not in others. During our research, we pooled the data on the association between temperature and dengue globally and found that dengue transmission would be accelerated with increasing temperature in the studied ranges, including minimum temperature, mean temperature and maximum temperature. Minimum temperature was critical in many regions for mosquito survival and development rate in sustaining the population density [12]. Low minimum temperatures had a negative effect on the survival of adult and immature *Aedes* mosquitoes, and higher minimum temperatures might assist larval survival in winter [14]. For instance, if monthly mean temperature fell to 16.5 °C, oviposition declined rapidly, and no eggs were produced if the mean temperature decreased to 14.8 °C in Buenos Aires, Argentina [11]. Additionally, if the annual mean temperature was higher than 11 °C, the mean temperature of the coldest month, January, higher than −5 °C, would be optimal for the existence of *Aedes albopictus* [13]. In contrast, a temperature above 18.1 °C was an inducing factor for a dengue epidemic, and the minimum temperatures in our study from 18.1 °C to 24.2 °C were positively associated with dengue risk (OR and 95% CI was 35.0% per 1 °C, 18.3%–51.6%).

The life cycle of *Aedes* mosquito is directly influenced by ambient environmental factors [57]. Increased temperature could improve DF transmission by increasing the development rate of mosquitoes and shortening virus incubation time in the body of mosquitoes [9], but extremely hot weather would impose restrictions on dengue transmission [17]. The maximum temperatures in the included articles ranged from 26 °C to 34.35 °C. Figure 1 shows that temperature was positively associated with DF incidence or cases, but from 29 °C, the OR of dengue risk began to decline. From Table 1, the mean temperature and the maximum temperature were still positively associated with DF, but the pooled OR of the maximum temperature was less than the mean temperature. Based on Watts experiments in Bangkok, a marked reduction in virus transmission rates in the extrinsic incubation period at 32 °C and an interruption of virus replication and dissemination to salivary glands was found [10]. Yang *et al.* [58] showed that during the interval 15 < T < 30 °C, female mosquitoes in the aquatic phase have a lower mortality rate, higher transition rate and higher offspring number, and Tun-Lin *et al.* [59] found that between 20 °C to 30 °C, the mosquitoes have a maximum survival rate of 88%–93%. In southern Taiwan, maximum temperature was negatively associated with DF incidence [60] because temperatures in this region often exceed the optimal temperature range of survival for adult mosquitoes (>30 °C) . Therefore, approximately 29 °C might be the inflexion of the upper limit for DF transmission influenced by temperature, which is consistent with our results and indicates that dengue risk would decrease if temperatures rise above 29 °C.

It has been reported that the critical minimum temperature suitable for DF transmission throughout the year was 21 °C if the daily survival rate of *Aedesagypti* was 0.89 in Hainan Province, China [15]. Nevertheless, our results indicate that 22 °C might be the lower limit at which DF incidence risk, as influenced by temperature, increases significantly. With global warming, the minimum temperatures rise at a faster rate than the maximum temperatures [61] especially in temperate and frigid zones. This factor would affect the activity of mosquitoes in many ways so that subtropical regions would be more suitable for DF transmission and DF would emerge in the temperate zones in the future. Understanding the risk of introducing dengue to non-infected areas is lacking in risk predictive models, and our results remind the government to evaluate the predictive capability of climate variables and their operational utility for surveillance.

Many studies have emphasized the importance of delayed effects on daily, weekly and monthly scales. In our study, we regarded delayed effect as a heterogeneity factor, but we did not find any difference between lag times, as there might be limited sample sizes in the subgroups. However, the delayed effect (or time lag) of meteorological variables on dengue transmission could be explained by meteorological factors that do not directly influence the incidence of dengue but indirectly influence transmission through their effects on the life cycle dynamics of the mosquito vector and virus [6]. Therefore, we suggest that researchers set a standardized scale for lag time, in addition to considering the conditions of the studied regions, such as a week or month for comparison purposes in future studies.

There are several limitations to our study that should be considered. First, we found significant heterogeneity across all temperatures, and the differences in lag time, statistical methods, and the altitude and climatic zone of studied regions might account for this heterogeneity. However, we did not find strong evidence for any of these or other factors. The limited sample size affected this heterogeneity analysis. Second, we report the estimation for temperatures, which does not consider the potential interactions of multiple meteorological factors or adjustments for collinearity [62,63]. Therefore, such potential interactions should be the focus of future study. Thirdly, there are inconsistent reports on secondary outcomes, which confine the analysis.

A geographic extension of dengue transmission regions has been projected as a consequence of climate change. Dengue risk will be more serious in the next few decades due to global warming unless effective and affordable vaccines or other interventions are made available soon [64]. More high-quality studies are urgently needed to establish the associations of other meteorological factors with dengue incidence. Accordingly, an early-warning system or long-term projection model should be established to prevent and control dengue transmission and outbreaks.

Most of the evidence demonstrates that dengue epidemics are closely related to temperature: 22 °C and 29 °C might be the critical range for DF transmission influenced significantly by temperatures. According to our systematic review, we suggest that government decision-makers and public health workers escalate DF control and prevention if the temperature rises to the interval between 22 °C to 29 °C. Due to global warming, a growth in temperature does not necessarily involve increasing the dengue incidence in tropical regions, but rather in subtropical and temperature zones, and the distribution and the seasonal duration would expand. Additionally, new epidemic regions would emerge. The distribution of dengue has already expanded to higher latitude and elevation areas. Due to a lack of knowledge about the disease, and because of inadequate measures and resources for prevention and control, there is likely to be an increase in the incidence and extent of outbreaks. Therefore, the departments for dengue prevention and control should consider global travel networks and climate change to adjust their strategies through mosquito surveillance and temperature observation, and to promote early prevention as a cost-effective and crucial strategy for maintaining public health worldwide.

## 5. Conclusions

This study aimed to quantitatively assess the influence of temperature on dengue incidence or global cases. Minimum temperature, mean temperature and maximum temperature were positively connected with dengue transmission based on the results obtained through systematic review and meta-analysis of the obtained data, and temperatures have the closest association with dengue in the range from 22 °C to 29 °C. Public health departments should adjust their dengue prevention and control strategies to account for changing temperatures, altered distribution ranges and different epidemic modes of dengue.

## Supplementary Files

Supplementary File 1
