# Supplementary material for: A Systematic Review and Meta-Analysis of Dengue Risk with Temperature Change
Source: Int J Environ Res Public Health. 2014 Dec 23;12(1):1–15. doi: 10.3390/ijerph120100001 (PMC4306847; doi:10.3390/ijerph120100001)
Supplement: Supplementary File 1 [file ijerph-12-00001-s001.pdf]

Supplementary Information

A Systematic Review and Meta-Analysis of Dengue Risk with Temperature Change

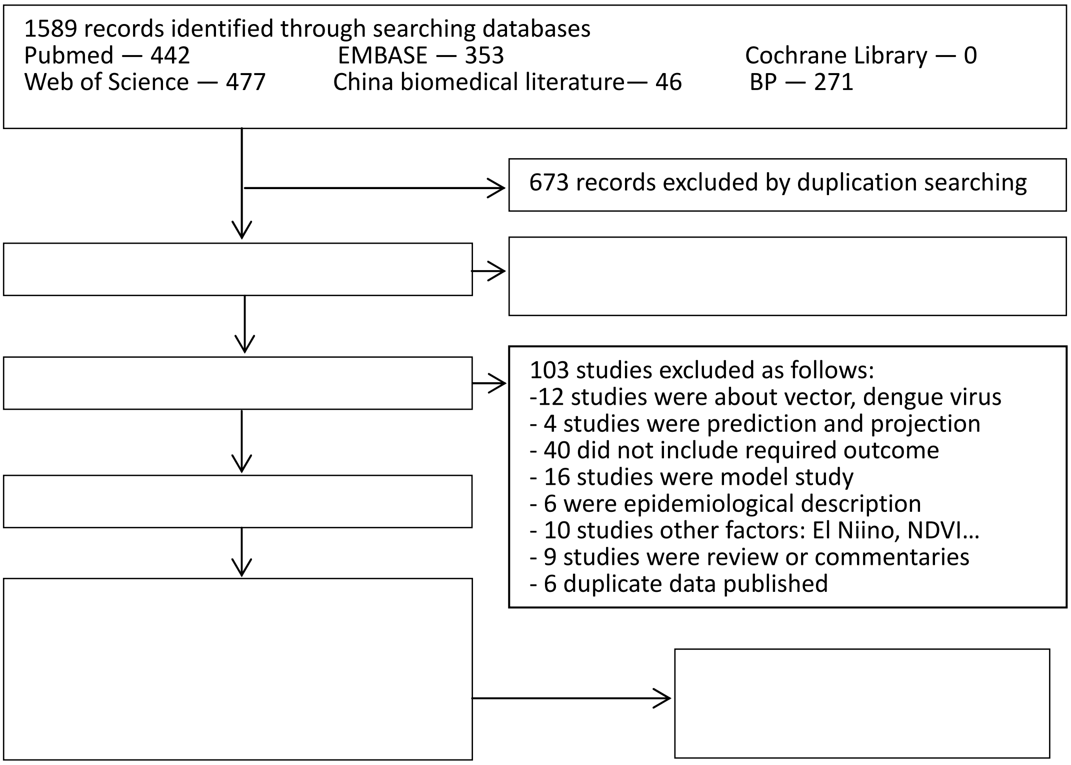

Figure S1. Flowchart of literature research and review process.

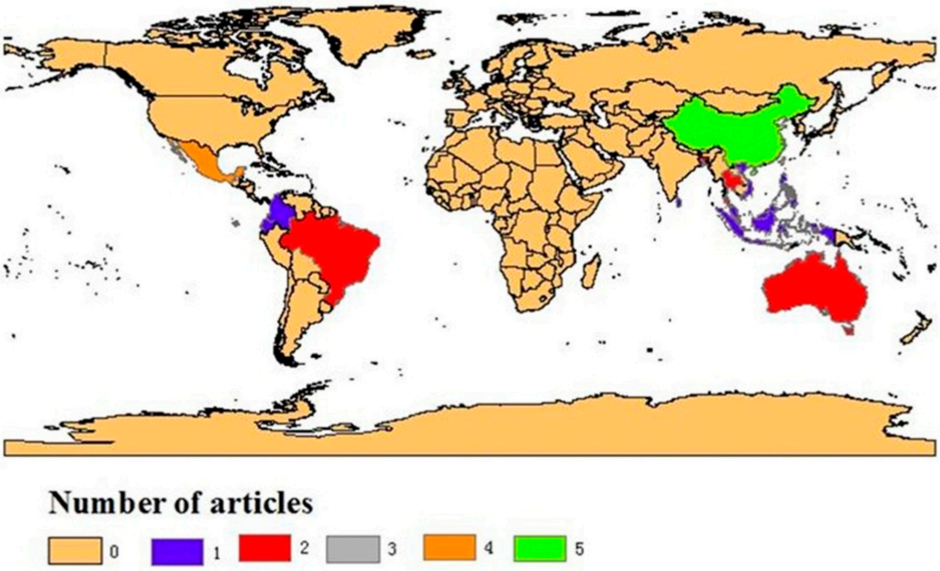

Figure S2. The location of the included articles.

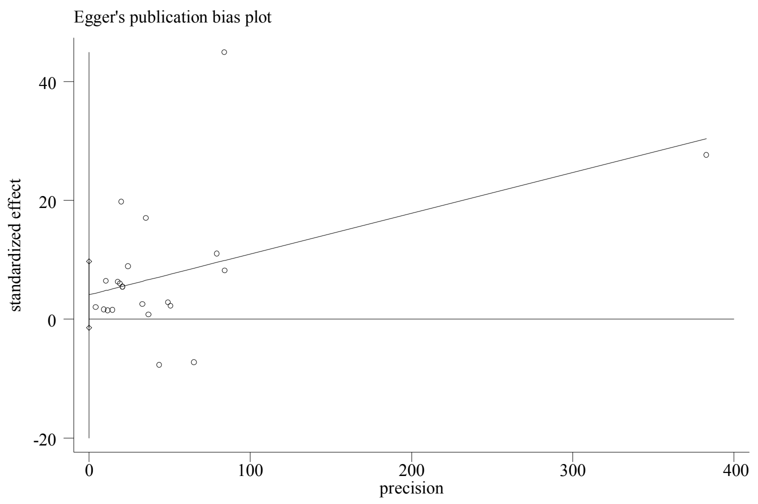

T_{min}

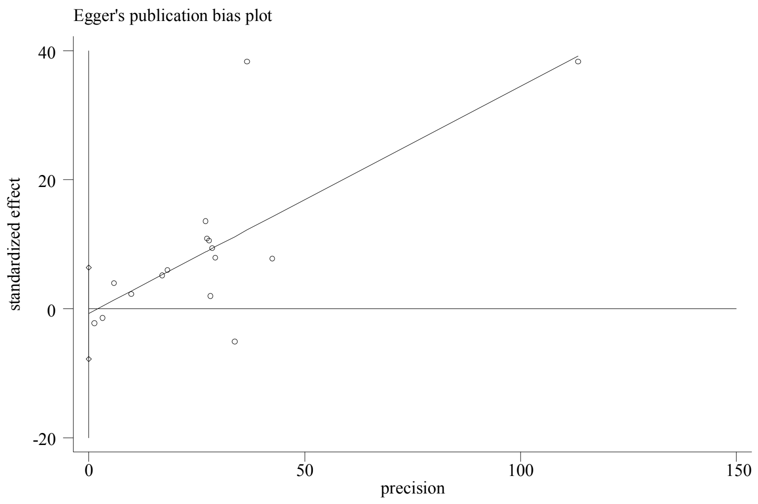

T_{mean}

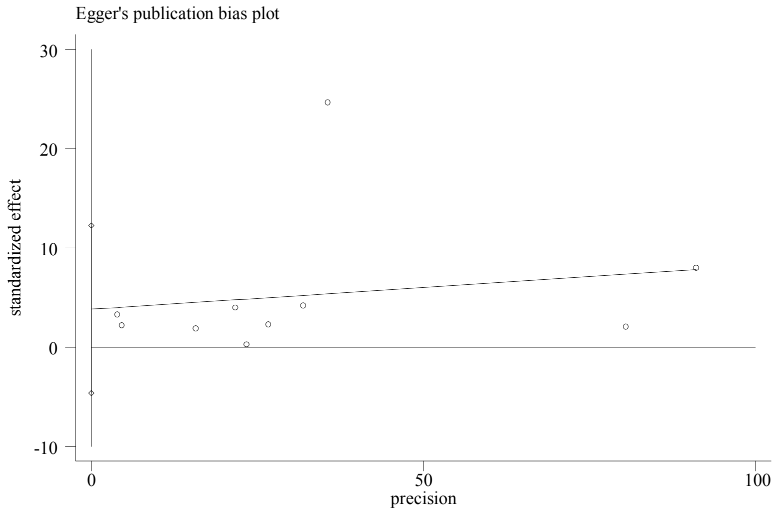

T_{max}

Figure S3. Egger's publication bias plot of the included articles.

Table S1. Contextual details of studies included in the meta-analysis by publication year.

No.	First Author	Published	Location	Period	Altitude (m)	Climatic Zone	Data Source of Dengue	Data Source of Meteorology	Statistical Method	Studied Variable	Measure of Effect	Outcome
[1]	Wu	2009	Taiwan	1998–2006	10	Tropical	NID Notification Surveillance System	Central Weather Bureau in Taiwan	Multiple logistic regression	*Tmean*	OR	Incidence rate
[2]	Lu	2009	Guangzhou	2001–2006	15	Subtropical	Chinese Center for Disease Control and Prevention	China Meteorological Data Sharing Service System	Multivariate Poisson regression	*Tmin*	*β*	Case
[3]	Yang	2009	Cixi, China	2004.7–2010	23	Subtropical	Zhejiang Provincial Center for Disease Control and Prevention	Archives of Meteorology, China Meteorological Administration	N/A	*Tmin Tmax Tmean*	*p*	Incidence rate
[4]	Rua-Uribe	2013	Medellin, Colombia	2001–2011	1500	Tropical	Unidades Primarias Generadoras de Datos	Meteorología y Estudios Ambientales de Colombia	Autoregressive integrated moving average	*Tmin, Tmax, Tmean*	*p*	Incidence rate
[5]	Depradine	2004	Barbados	1995–2000	10	Tropical	N/A	N/A	Multiple linear regression	*Tmin Tmax Tmean*	*p*	Case
[6]	Promprou	2005	Southern Thailand	1993–2002	38	Tropical	Ministry of Public Health	Climatology Division of the Meteorological Department	Multiple linear regression	*Tmin Tmax Tmean*	*β*	Incidence rate
[7]	Chowell	2006	Colima, Mexico	2002	482	Tropical	Secretariat of Public Health	Local meteorological offices	Multiple linear regression	*Tmin Tmax Tmean*	*β*	Incidence rate
[8]	Hurtado-Daz	2006	Veracruz, Mexico	1995–2003	10	Tropical	Veracruz State Health Ministry	National Meteorological Service and Regional Gulf-Central Administration	Second-order polynomial regression	*Tmin*	*β*	Incidence rate
[9]	Arcari	2007	Indonesia	1992–2001	16	Tropical	Indonesian Ministry of Health	Bureau of Meteorology in Jakarta	Multiple linear regression	*Tmean*	*p*	Incidence rate
[10]	Brunkard	2008	Matamoros, Tamaulipas	1995–2005	1	Subtropical	Matamoros, Tamaulipas, Mexico	National Climatic Data Center	Multiple linear regression	*Tmax*	OR	Incidence rate
[11]	Su	2008	Metro Manila Philippines	1996–2005	13	Tropical	National Epidemiology Sentinel Surveillance System	Philippine Atmospheric, Geophysical, and Astronomical Services	Multiple linear regression	*Tmean*	*p*	Incidence rate

Table S1. *Cont.*

No.	First Author	Published	Location	Period	Altitude (m)	Climatic Zone	Data Source of Dengue	Data Source of Meteorology	Statistical Method	*Studied Variable*	Measure of Effect	Outcome
[12]	Hsieh	2009	Taiwan	e-weeks 23–52, 2007	10	Tropical	Taiwan Centers for Disease Control	N/A	Richards model	*Tmin Tmax Tmean*	OR	Case/ Inci-dence rate
[13]	Chen	2010	Taiwan	2001–2008	10	Tropical	Taiwan Centers for Disease Control	Taiwan Environmental Protection Agency	Multivariate Poisson regression	*Tmin*	*β*	Incidence rate
[14]	Sriprom	2010	Sakon Nakhon Province, Thailand	2005–2007	187	Tropical	Sakon Nakhon Provincial Public Health Office	Sakon Nakhon Meteorological Office	Generalized Linear Mode	*Tmin*	*β*	Incidence rate
[15]	Colon-González	2011	Warm and humid provinces of Mexico	1985–2007	580	Tropical	National System of Epidemiologic Surveillance in Mexico	Mexican National Meteorological Service	Multiple linear regression	*Tmin Tmax*	*β*	Cumulative incidence rate
[16]	Gharbi	2011	Guadeloup, French West Indies	2000–2007	59	Tropical	Institut Pasteur in Guadeloupe	The meteorological station of Raizet	Seasonal Autoregressive Integrated Moving Average	*Tmin, Tmean*	*β*	Incidence rate
[17]	Lai	2011	Kaohsiung, Taiwan	2002–2007	9	Tropical	Taiwan Centers for Disease Control	Taiwan Environmental Protection Administration and Central Weather Bureau	Duncan's Multiple Range test	*Tmin Tmax*	*p*	case
[18]	Pham	2011	Dak Lak, Vietnam	2004–2008	600	Tropical	Province's Center for Preventive Health	Dak Lak meteorological office	Multivariate Poisson regression	*Tmean*	*β*	Incidence rate
[19]	Pinto	2011	Singapore	2000–2007	23	Tropical	Ministry of Health Singapore	Climate Diagnostic Center and National Oceanographic and Atmospheric Administration	Multivariate Poisson regression	*Tmin, Tmax*	*β*	Incidence rate

Table S1. *Cont.*

No.	First Author	Published	Location	Period	Altitude (m)	Climatic Zone	Data Source of Dengue	Data Source of Meteorology	Statistical Method	*Studied Variable*	Measure of Effect	Outcome
[20]	Earnest	2012	Singapore	2001–2008	23	Tropical	Ministry of Health Singapore	Meteorological Services Division of the National Environment Agency	Multivariate Poisson regression	*Tmean*	*β*	Incidence rate
[21]	Gomes	2012	Rio de Janeiro, Brazil	2001–2009	67	Tropical	Rio de Janeiro City Health Department	Weather Forecasting Center of the National Space Research Institute	Binomial negative Poisson regression	*Tmin*	OR	Case
[22]	Hashizume	2012	Dhaka, Bangladesh	2005–2009	23	Tropical	11 principal hospitals in the Dhaka Metropolitan area	Bangladesh Meteorological Department	Generalized linear Poisson regression	*Tmin Tmax Tmean*	OR	Case
[23]	Hii	2012	Singapore	2000–2010	23	Tropical	Ministry of Health Singapore	Climatic Data Center, National Oceanic and Atmospheric Administration	Multivariate Poisson regression	*Tmean*	OR	Case
[24]	Hu	2012	Queenslan, Australia	2003–2005	130	Tropical	Queensland Health Department	Australian Bureau of Meteorology	Separate Poisson regression models	*Tmax*	OR	Case
[25]	Karim	2012	Dhaka, Bangladesh	2000–2007	23	Tropical	Diseases Control Room of Directorate General of Health Services	Bangladesh Meteorological Department at Dhaka	Multiple linear regression	*Tmean*	*β*	Case
[26]	Cheong	2013	Malaysia	2008–2010	56	Tropical	Ministry of Health Malaysia	National Climatic Data Center website	Poisson generalized additive models	*Tmin, Tmax*	OR	Case
[27]	Goto	2013	Sri Lanka	2005–2011	54	Tropical	Ministry of Health, Sri Lanka	Department of Meteorology in Sri Lanka	The ordinary least squares	*Tmean*	*β*	Incidence rate
[28]	Huang	2013	Cairns, Australia	2000–2009	8	Tropical	Queensland Health Department	Australian Bureau of Meteorology	Negative-binomial hurdle model	*Tmin*	OR	Case
[29]	Li	2013	Guangzhou, China	2007–2012	15	Subtropical	N/A	N/A	Negative binomial regression	*Tmin*	OR	Incidence rate
[30]	Lowe	2013	Southeast Brazil	2001–2009	392	Tropical	DATASUS (http://dtr2004.saude.gov.br/sinanweb/novo/)	Global Precipitation Climatology Project	Bayesian general linear mixed model	*Tmean*	*β*	Incidence rate

Table S1. *Cont.*

No.	First Author	Published	Location	Period	Altitude (m)	Climatic Zone	Data Source of Dengue	Data Source of Meteorology	Statistical Method	*Studied Variable*	Measure of Effect	Outcome
[31]	Stewart-Ibarra	2013	El Oro, Ecuador	1995–2010	6	Tropical	Ministry of Health Ecuador	National Institute of Meteorology and Hydrology	Generalized linear mixed model	*Tmin*	OR	Standardized morbidity ratios
[32]	Fan	2013	Guangdong, China	2005–2011	15	Subtropical	National Noticeable Disease Surveillance System	China Meteorological Data Sharing Service System	Time-stratified case-crossover model	*Tmin Tmax Tmean*	OR	Case
[33]	Wang	2013	Guangzhou, China	2000–2012	15	Subtropical	Chinese Center for Disease Control and Prevention	China Meteorological Data Sharing Service System	Zero-inflated Poisson Regression Model	*Tmin*	*β*	Case

Note: N/A = not applicable.

Table S2. Quality assessment of selected studies.

Author	Year	Generalizability	Description of Temperature	Dengue Cases or Incidence	Source of Dengue Data	Reporting Bias	Limitation	Multiple Lag	Adjusted for Time Trend	Adjusted for Seasonality	Fund Supporting
Wu *et al.* [1]	2009	1	1	1	1	1	1	1	1	1	0
Lu *et al.* [2]	2009	1	1	1	1	1	0	1	0	0	1
Yang *et al.* [3]	2009	0	1	1	1	1	1	N/A	N/A	N/A	1
Rua-Uribe *et al.* [4]	2013	1	1	1	1	1	0	1	0	0	0
Depradine *et al.* [5]	2004	1	0	1	1	1	0	1	1	0	0
Promprou *et al.* [6]	2005	1	1	1	1	1	0	0	0	0	1
Chowell *et al.* [7]	2006	1	1	1	1	1	1	1	1	1	0
Hurtado-Daiz *et al.* [8]	2006	1	1	1	1	1	1	1	1	1	0
Arcari *et al.* [9]	2007	1	1	1	1	1	1	1	0	1	0
Brunkard *et al.* [10]	2008	1	1	1	1	1	1	1	0	0	0
Su [11]	2008	1	0	1	1	1	0	0	0	0	0
Hsieh *et al.* [12]	2009	1	1	1	1	1	0	1	1	0	1

Table S2. *Cont.*

Author	Year	Generalizability	Description of Temperature	Dengue Cases or Incidence	Source of Dengue Data	Reporting Bias	Limitation	Multiple Lag	Adjusted for Time Trend	Adjusted for Seasonality	Fund Supporting
Chen *et al.* [13]	2010	1	1	1	1	1	1	1	0	0	0
Sriprom *et al.* [14]	2010	1	1	1	1	1	0	0	0	0	0
Colon- González *et al.* [15]	2011	1	1	1	1	1	1	1	1	1	1
Gharbi *et al.* [16]	2011	1	1	1	1	1	1	1	1	1	0
Lai [17]	2011	1	1	1	1	1	0	1	0	0	0
Pham *et al.* [18]	2011	1	1	1	1	1	1	0	0	0	0
Pinto *et al.* [19]	2011	1	1	1	1	1	1	1	0	0	0
Earnest *et al.* [20]	2012	1	1	1	1	1	1	1	1	1	1
Gomes *et al.* [21]	2012	1	1	1	1	1	1	1	0	1	0
Hashizume *et al.* [22]	2012	1	1	1	1	1	1	1	1	1	1
Hii *et al.* [23]	2012	1	1	1	1	1	1	1	0	0	1
Hu *et al.* [24]	2012	1	1	1	1	1	1	0	0	0	1
Karim *et al.* [25]	2012	1	1	1	1	1	0	0	1	1	0
Cheong *et al.* [26]	2013	1	1	1	1	1	0	1	1	1	0
Goto *et al.* [27]	2013	1	1	1	1	1	1	1	0	0	1
Huang *et al.* [28]	2013	1	1	1	1	1	1	1	0	1	1
Li *et al.* [29]	2013	1	1	1	0	1	1	1	0	0	1
Lowe *et al.* [30]	2013	1	1	1	1	1	1	1	1	1	1
Stewart-Ibarra *et al.* [31]	2013	1	1	1	1	1	0	1	0	0	1
Fan *et al.* [32]	2013	1	1	1	1	1	1	1	1	1	1
Wang *et al.* [33]	2013	1	1	1	1	1	1	1	0	0	1

Notes: N/A = not applicable; 1 = yes; 0 = no.

References

1. Wu, P.C.; Lay, J.G.; Guo, H.R.; Lin, C.Y.; Lung, S.C.; Su, H.J. Higher temperature and urbanization affect the spatial patterns of dengue fever transmission in subtropical Taiwan. *Sci. Total Environ.* **2009**, *407*, 2224–2233.
2. Lu, L.; Lin, H.L.; Tian, L.W.; Yang, W.Z.; Sun, J.M.; Liu, Q.Y. Time series analysis of dengue fever and weather in Guangzhou, China. *BMC Public Health* **2009**, *9*, 395–399.
3. Yang, T.C.; Lu, L.; Fu, G.M.; Zhong, S.; Ding, G.Q.; Xu, R.; Zhu, G.F.; Shi, N.F.; Fan, F.L; Liu, Q.Y. Epidemiology and vector efficiency during a dengue fever outbreak in Cixi, Zhejiang Province, China. *J. Vector Ecol.* **2009**, *34*, 148–154.
4. Rua-Uribe, G.L.; Suarez-Acosta, C.; Chauca, J.; Ventosilla, P.; Almanza, R. Modelling the effect of local climatic variability on dengue transmission in Medellin (Colombia) by means temporary series analysis. *Biomedica* **2013**, *33*, 142–152.
5. Depradine, C.A.; Lovell, E.H. Climatological variables and the incidence of dengue fever in Barbados. *Int. J. Environ. Health Res.* **2004**, *14*, 429–441.
6. Promprou, S.; Jaroensutasinee, M.; Jaroensutasinee, K. Climatic factors affecting dengue haemorrhagic fever incidence in southern Thailand. *Dengue Bull.* **2005**, *29*, 41–48.
7. Chowell, G.; Sanchez, F. Climate-based descriptive models of dengue fever: The 2002 epidemic in Colima, Mexico. *J. Environ. Health* **2006**, *68*, 40–44.
8. Hurtado-Daiz, M.; Riojas-Rodrguez, H.; Rothenberg, S.; Gomez-Dantes, H.; Cifuentes, E. Impact of climate variability on the incidence of dengue in Mexico. *Trop. Med. Int. Health* **2007**, *12*, 1327–1337.
9. Arcari, P.; Tapper, N.; Pfueller, S. Regional variability in relationships between climate and dengue/DHF in Indonesia. *Singap. J. Trop. Geogr.* **2007**, *28*, 251–272.
10. Brunkard, J.M.; Cifuentes, E.; Rothenberg, S.J. Assessing the roles of temperature, precipitation, and ENSO in dengue re-emergence on the Texas-Mexico border region. *Salud. Publ. Mex.* **2008**, *50*, 227–234.
11. Su, G. L. Correlation of climatic factors and dengue incidence in Metro Manila, Philippines. *Ambio* **2008**,*37*, 292–294.
12. Hsieh, Y.H.; Chen, C.W. Turning points, reproduction number, and impact of climatological events for multi-wave dengue outbreaks. *Trop. Med. Int. Health* **2009**, *14*, 628–638.
13. Chen, S.C.; Liao, C.M.; Chio, C.P.; Chou, H.H.; You, S.H.; Cheng, Y.H. Lagged temperature effect with mosquito transmission potential explains dengue variability in southern Taiwan: Insights from a statistical analysis. *Sci. Total Environ.* **2010**, *408*, 4069–4075.
14. Sriprom, M.; Chalvet-Monfray, K.; Chaimane, T.; Vongsawat, K.; Bicout, D.J. Monthly district level risk of dengue occurrences in Sakon Nakhon Province, Thailand. *Sci. Total Environ.* **2010**, *408*, 5521–5528.
15. Colon-Gonzalez, F.J.; Lake, I.R.; Bentham, G. Climate variability and dengue fever in warm and humid Mexico. *Amer. J. Trap. Med. Hyg.* **2011**, *84*, 757–763.
16. Gharbi, M.; Quenel, P.; Gustave, J.; Cassadou, S.; Ruche, G.L.; Girdary, L.; Marrama, L. Time series analysis of dengue incidence in Guadeloupe, French West Indies: Forecasting models using climate variables as predictors. *BMC Infect. Dis.* **2011**, *11*, doi:10.1186/1471-2334-11-166.

17. Lai, L.W. Influence of environmental conditions on asynchronous outbreaks of dengue disease and increasing vector population in Kaohsiung, Taiwan. *Int. J. Environ. Health Res.* **2011**, *21*, 133–146.
18. Pham, H.V.; Doan, H.T.; Phan, T.T.; Minh, N.N. Ecological factors associated with dengue fever in a Central Highlands province, Vietnam. *BMC Infect. Dis.* **2011**, *11*, doi:10.1186/1471-2334-11-172.
19. Pinto, E.; Coelho, M.; Oliver, L.; Massad, E. The influence of climate variables on dengue in Singapore. *Int. J. Environ. Health Res.* **2011**, *21*, 415–426.
20. Earnest, A.; Tan, S.B.; Wilder-Smith, A. Meteorological factors and El Niño southern oscillation are independently associated with dengue infections. *Epidemiol. Infect.* **2012**, *140*, 1244–1251.
21. Gomes, A.F.; Nobre, A.A.; Cruz, O.G. Temporal analysis of the relationship between dengue and meteorological variables in the city of Rio de Janeiro, Brazil, 2001–2009. *Cad. Saude. Publ.* **2012**, *28*, 2189–2197.
22. Hashizume, M.; Dewan, A.M.; Sunahara, T.; Rahman, M.Z.; Yamamoto, T. Hydroclimatological variability and dengue transmission in Dhaka, Bangladesh: A time-series study. *BMC Infect. Dis.* **2012**, *12*, doi:10.1186/1471-2334-12-98.
23. Hii, Y.L.; Rocklov, J.; Wall, S.; Ng, L.C.; Tang, C.S.; Ng, N. Optimal lead time for dengue forecast. *PLoS Negl. Trop. Dis.* **2012**, *6*, doi:10.1371/journal.pntd.0001848.
24. Hu, W.B.; Clements, A.; Williams, G.; Tong, S.L.; Mengersen, K. Spatial patterns and socioecological drivers of dengue fever transmission in Queensland, Australia. *Environ. Health Perspect.* **2012**, *120*, 260–266.
25. Karim, M.N.; Munshi, S.U.; Anwar, N.; Alam, M.S. Climatic factors influencing dengue cases in Dhaka city: A model for dengue prediction. *Indian J. Med. Res.* **2012**, *136*, 32–39.
26. Cheong, Y.L.; Burkart, K.; Leitão, P.J.; Lakes, T. Assessing weather effects on dengue disease in Malaysia. *Int. J. Environ. Res. Public Health.* **2013**, *10*, 6319–6334.
27. Goto, K.; Kumarendran, B.; Mettananda, S.; Gunasekara, D.; Fujii, Y.; Kaneko, S. Analysis of effects of meteorological factors on dengue incidence in Sri lanka using time series data. *PLoS One* **2013**, *8*, doi:10.1371/journal.pone.0063717.
28. Huang, X.; Williams, G.; Clements, A.C.; Hu, W. Imported dengue cases, weather v ariation and autochthonous dengue incidence in Cairns, Australia. *PLoS One* **2013**, *8*, doi:10.1371/journal.pone.0081887.
29. Li T.G.; Yang Z.C.; Luo, L.; Di, B.; Wang, M. Dengue fever epidemiological status and relationship with meteorological variables in Guangzhou, southern China, 2007–2012. *Biomed. Environ. Sci.* **2013**, *26*, 994–997.
30. Lowe, R.; Bailey, T.C.; Stephenson, D.B.; Jupp, T.E.; Graham, R.J.; Barcellos, C.; Carvalho, M.S. The development of an early warning system for climate-sensitive disease risk with a focus on dengue epidemics in southeast Brazil. *Stat. Med.* **2013**, *32*, 864–883.
31. Stewart-Ibarra, A.M.; Lowe, R. Climate and non-climate drivers of dengue epidemics in southern coastal Ecuador. *Amer. J. Trap. Med. Hyg.* **2013**, *88*, 971–981.
32. Fan, J.C.; Lin, H.L.; Wang, C.G.; Bai, L.; Yang, S.R; Chu, C.; Yang, W.Z; Liu, Q.Y. Identifying the high-risk areas and associated meteorological factors of dengue transmission in Guangdong Province, China from 2005 to 2011. *Epidemiol. Infect.* **2014**, *142*, 634–643.

33. Wang, C.G.; Jiang, B.F.; Fan, J.C.; Wang, F.R.; Liu, Q.Y. A study of the dengue epidemic and meteorological factors in Guangzhou, China, by using a zero-inflated poisson regression model. *Asia Pac. J. Public Health* **2014**, *26*, 48–57.

© 2014 by the authors; licensee MDPI, Basel, Switzerland. This article is an open access article distributed under the terms and conditions of the Creative Commons Attribution license (http://creativecommons.org/licenses/by/4.0/).
